# An integrative modeling approach to the age-performance relationship in mammals at the cellular scale

**DOI:** 10.1038/s41598-018-36707-3

**Published:** 2019-01-23

**Authors:** Geoffroy Berthelot, Avner Bar-Hen, Adrien Marck, Vincent Foulonneau, Stéphane Douady, Philippe Noirez, Pauline B. Zablocki-Thomas, Juliana da Silva Antero, Patrick A. Carter, Jean-Marc Di Meglio, Jean-François Toussaint

**Affiliations:** 1 Institut de Recherche bio-Médicale et d’Epidémiologie du Sport (IRMES), EA 7329, Institut National du Sport de l’Expertise et de la Performance (INSEP) and Université Paris Descartes, Sorbonne Paris Cité, Paris, France; 2 REsearch LAboratory for Interdisciplinary Studies (RELAIS), Paris, France; 3 CNAM, 75003 Paris, France; 4 Laboratoire Matière et Systèmes Complexes, UMR 7057, Université Paris Diderot and CNRS, Sorbonne Paris Cité, Paris, France; 5 Département de Biologie, ENS de Lyon, Lyon, France; 6 Département d’écologie et de Gestion de la Biodiversité, UMR 7179 CNRS/MNHN, Paris, France; 7 School of Biological Sciences, Washington State University, Pullman, USA; 8 CIMS, Hôtel-Dieu, APHP, Paris, France

## Abstract

Physical and cognitive performances change across lifespan. Studying cohorts of individuals in specific age ranges and athletic abilities remains essential in assessing the underlying physiological mechanisms that result in such a drop in performance. This decline is now viewed as a unique phenotypic biomarker and a hallmark of the aging process. The rates of decline are well documented for sets of traits such as running or swimming but only a limited number of studies have examined the developmental and senescent phases together. Moreover, the few attempts to do so are merely descriptive and do not include any meaningful biological features. Here we propose an averaged and deterministic model, based on cell population dynamics, replicative senescence and functionality loss. It describes the age-related change of performance in 17 time-series phenotypic traits, including human physical and cognitive skills, mouse lemur strength, greyhound and thoroughbred speed, and mouse activity. We demonstrate that the estimated age of peak performance occurs in the early part of life (20.5% ± 6.6% of the estimated lifespan) thus emphasizing the asymmetrical nature of the relationship. This model is an initial attempt to relate performance dynamics to cellular dynamics and will lead to more sophisticated models in the future.

## Introduction

Pierre de Coubertin revived the Olympic Games in 1896. Since then, international sport competitions have become major events which make evident the progression of human performances over the years^1^. Practical tools have been progressively developed to measure human speed and stamina and to explore the underlying physiology of these performance traits^2–4^. The fast pace of technological innovations now allows for a precise measurement of human performance, such as the top speeds in running events. These measurements extend to other species used in sport, such as greyhounds and thoroughbreds^5,6^. The very large amount of recorded data now allows for the investigation of key questions such as the presence of physical limitations^1,5,6^. Performance depends on numerous factors, including genetics^7,8^, environment (such as ambient temperature)^1^ and/or technology^1^. A leading determinant of performance is the chronological age of the athlete^9,10^. In humans, decline in physical performance usually occurs by age 20–30^9–12^, as does working capacity^13,14^ and other physiological abilities^15–20^. Knechtle *et al*. showed exceptions to this rule; for example, the age-related performance decline starts later in life for running long distances, possibly because of experience^20,21^. Increased reaction time, decreased coordination and joint mobility, decreased skeletal size and muscle bulk, decreased type 2 fast-twitch muscle fibers, changed body fat composition, and decreased cardiovascular and respiratory functions are among the physiological factors that are associated with the observed decline^11,22,23^. Belsky *et al*. also showed an increasing pace of coordinated deterioration across multiple organ systems with an exponential increase in physical burden (e.g. pain, fatigue) from several different chronic conditions after the fifth decade of life^24^. The rate of physical decline in aging adults has been studied in various disciplines^10–12,25^ and is now regarded as a hallmark of the aging process^4,26^, and serves as a key indicator of diminishing quality of life while remaining a predictor of physical disability and other morbidities^4^. Justice *et al*. argued that functional assessments provide a unique and practical phenotypic biomarker, as well as a convenient tool to measure response to later life interventions^4^.

From a broader perspective, the complete lifetime trajectory from young to advanced ages was investigated by Dan H. Moore for track and field performances^27^. He showed that the age-related changes of top physical performance in 15 running and two throwing events exhibit an inverted-U pattern that can be described using a single equation based on two non-linear functions:

1 $$P(t)=a(1-{e}^{-bt})+c(1-{e}^{dt}),\,P(t)\ge 0$$

where *a*, *b*, *c*, *d* are four positive constants and *P*(*t*) is the performance value at age *t*. Equation (1) can be accurately applied to existing datasets in a variety of species^10,25,26^ for a number of different individual athletic or cognitive capacities despite inter-individual variability^10,25^. Other studies have used second-order polynomial equations to fit the pattern^9^; however, the age-performance relationship is consistently reported to be asymmetrical, with an ‘early’ (*i*.*e*. before mid lifespan) age of peak performance^10,25–27^, meaning that quadratic functions, such as second order polynomials, provide a poor estimate of the age of peak performance.

## Working at The Cellular Scale

The equations used to describe the age-related physical performance, such as Eq. (1), are designed from a statistical and empirical perspective and do not include any biological or physiological assumption(s) in their approach (e.g., the four constants in Eq. (1) do not have a particular biological meaning). In fact, excluding biological assumptions often leads to peculiar equations, such as Eq. (1) that separates the growing (maturation) from the declining (aging) process. Moreover, such relationships must be driven by developmental changes and senescence in physiological systems. Muscle mass, body mass and height, oxygen uptake, hormones and other physiological parameters progressively change with age to allow for an increase in performance from infancy to adulthood. Likewise, a functional decrease then progressively takes place, affecting such physiological factors as lung volume, muscle width, testosterone index, etc., resulting in declining performance at older ages^26^. Importantly, most of these physiological parameters are determined by mechanisms occurring at the cellular level. The cell is a fundamental biological unit of all known living organisms, suggesting that the cellular scale is useful for developing inter-specific models of age-related physical performance.

In terms of cell dynamics, population models can typically be used to describe cell replication, proliferation and death. In particular, the well-known Siler model^28^:

2 $$q(t)={a}_{1}.\,{e}^{-{b}_{1}t}+{a}_{2}+{a}_{3}.\,{e}^{{b}_{3}t}$$

describes mortality dynamics, with the total hazard function *q*(*t*) depending on age-related hazards *a*_1_,*b*_1_ (immature animals), *a*_2_,*b*_2_ (mature animals), *a*_3_,*b*_3_ (senescence). Interestingly, Eq. (2) becomes Eq. (1) -i.e. $q(t)={a}_{1}(1-{e}^{-{b}_{1}t})$ $+{a}_{3}(1-{e}^{{b}_{3}t})$- when the constant hazard for mature animals is changed to an age-dependent hazard with value ${a}_{2}\equiv {a}_{2}(t)={a}_{1}+{a}_{3}-2{a}_{1}{e}^{-{b}_{1}t}-2{a}_{3}{e}^{{b}_{3}t}$. This is similar to the Heligman and Pollard (HP) approach based on the typical Gompertz law for modeling age-related mortality^29^. Using Siler approach, parameters *a*, *b*, *c*, *d* of Eq. (1) can now be interpreted as the initial hazard for immature $({a}_{1}\equiv a)$ or mature $({a}_{3}\equiv c)$ animals and $1/{b}_{1,3}$ are the two time constants with which the immature $({b}_{1}\equiv b)$ and mature $({b}_{3}\equiv d)$ hazards are reduced. Both Siler and HP models include predation and a countable number of developmental phases in which hazards occur, limiting their scope of application. However, models that describe population dynamics provide a guide for the design of a bottom-up approach to the age-performance relationship of multiple species at the cellular level. In particular, the Siler and Moore analogy is interesting as it lays the foundations for defining a general model of lifetime changes in performance.

## Objectives

Here we aim to introduce a model that is a first step in describing the biological basis of the asymmetrical and inverted-U pattern typically seen in performance curves. The motivation is to link organismal performance to the elementary units on which it relies: cells. We will use a population approach to model the observed performance patterns while defining cells as the elementary component of the organism. This new model is designed to be simple and expandable. We test the model for a variety of physiological functions and species, including five terrestrial mammals (human, thoroughbred, greyhound, mouse, mouse lemur).

## Materials and Methods

We define performance *P*(*t*) as the measurable outcome of a given system. The measured speed of an individual in a 100 m track and field event, the crawling speed of a snail, the distance jumped by a frog, the lactation performance (*i*.*e*., milk production) of a cow are such examples of performance. We assume that the observed performance results from the contribution of all the elementary components to the system.

### General model of lifetime changes in performance

Consider a population of *N* cells that grow during the development phase. Population models, such as Eq. (2), provide some useful guides to handle the dynamics of *N:* first, we shall consider that the detrimental processes occurring with aging progressively appear with time in a continuous manner. Hazards in Siler & HP approaches are also continuously related to time through three different stages of life (as in Eq. (2)). Second, in Siler & HP approaches, hazards are additive and non-interacting. We choose a similar formalization for the preliminary model presented here because it is simpler to ignore interaction. We recognize that performance is a complex process influenced by both emergent physiological processes as well as cell traits. For the purposes of this approach, we chose to simplify the model and therefore focus on the cell scale because of the difficulty of modeling emergent processes.

The general equation governing the performance with aging *P*(*t*) in continuous time, with non-interacting cell types may then be written as:

3 $$\{\begin{array}{c}\frac{d{N}_{i}(t)}{dt}={\alpha }_{i}(t){N}_{i}(t)\\ P(t)=\sum _{i}{{\rm{\Phi }}}_{i}{\beta }_{i}(t){N}_{i}(t)\end{array}$$

where *N*_*i*_(*t*) is a population of cells of a given type *i* (neurons, specialized myocytes, etc.), *α*_*i*_(*t*) is the growth rate, $\sum _{i}{N}_{i}=N$, the total population of all cells, *β*_*i*_(*t*) is a senescence parameter that embeds the adverse biological effects as a simple rate of decline that appears with aging. Parameter *c* is the contribution of the population of cells to the observed performance *P*(*t*). For example, if the performance measured is speed in m.s^−1^, then Φ_*i*_ is the increase in speed per cell.

Empirical studies demonstrate the existence of heterogeneity in performance changes with aging, thus emphasizing that both the rate of increase *α*_*i*_(*t*) and decline *β*_*i*_(*t*) may differ among cell types^30^. Three issues arise when taking into account cell specialization:

- parameter Φ_*i*_ and functions *α*_*i*_(*t*), *β*_*i*_(*t*) remain unknown and depend on the cell type. However, it is possible to roughly infer which particular cell types may matter during a specific task.
- the regeneration/replacement effect that takes place in many cell types: dead cells are partly replaced by new ones, leading to a variation of *N*(*t*) at each time step. We assume that this effect is embedded in *β*_*i*_(*t*).
- Interactions may occur between different cellular types, leading to joint/cooperative contribution to the performance. As previously mentioned, we ignore interaction in Eq. (3) at this time.

Thus we have focused on a simpler and averaged approach, designed to offer an appropriate trade-off between tractability and complexity while efficiently describing the chosen time series from a biological perspective.

### Integrative model of age-performance (IMAP1)

In order to account for holistic biological effects, we next introduce the Integrative Model of Age Performance (IMAP1) that integrates the dynamics of all cell types simultaneously. This model will necessarily look very similar to Eq. (3). We focus on the quantity *N*(*t*) at time *t* and define *α*(*t*) and *β*(*t*) as two age-related parameters that start at the time of the first cellular division t* with *t* ≥ *t*_0_ > t* and *t*_0_ = 0 years. Parameters *α*(*t*) and *β*(*t*) are two decreasing functions of time. The first parameter is the growth rate of the population of cells that includes growth-limiting factors that progressively occur with maturation and limiting body size^31^ that result in a progressive decline in cell proliferation, leading to an asymptotic saturation of *N*(*t*). The second parameter *β*(*t*) is the averaged monotonic age-related rate of decline of functionality of all cells *N*(*t*). This is a fundamental process that occurs in all cell types irrespective of specific functionality. For example, metabolic wastes and protein aggregation are processes known to lead to decreased functionality in all cell types^32^; for instance, lipofuscin residues building up in cells are associated with detrimental effects on functionality in several organs^33^. These two parameters *α*(*t*) and *β*(*t*) capture the system behavior as a whole, integrating the dynamics of all cell types including their respective contributions Φ_*i*_. The model is formulated as:

4 $$\{\begin{array}{c}\frac{dN(t)}{dt}=\alpha (t)N(t)\\ P(t)=\beta (t)N(t)\end{array}$$

We can more specifically define *α*(*t*), the growth parameter, as

5 $$\alpha (t)={\alpha }_{0}{e}^{-{\alpha }_{r}t}$$

where ${\alpha }_{0}=\alpha ({t}_{0}) > 0$, the initial (or baseline) average value of the saturation (*i*.*e*. the value at *t*_0_), which corresponds to the average time of cellular division of *N*(*t*). Parameter *α*_*r*_ > 0 is the strength of saturation, which is the amount of time required to reach the asymptote of cell growth. Based on the known decline of performance with aging, we assume that *β*(*t*), the average age-related decline of cell functionality, has the following form:

6 $$\beta (t)={\beta }_{0}(1-{e}^{{\beta }_{r}(t-{t}_{d})})$$

**Figure 1:**
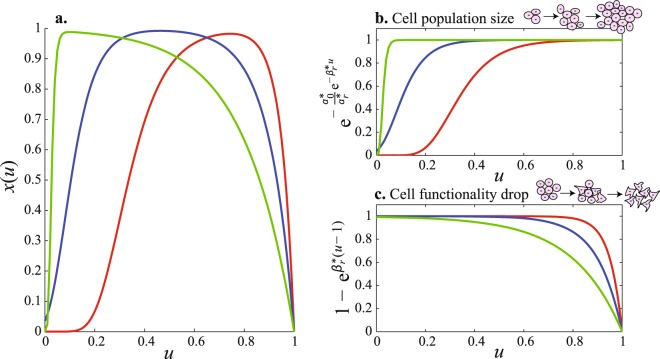
IMAP1 with three sets of parameters values. (**a**) Green line corresponds to ${\alpha }_{0}^{\ast }=1000$, ${\alpha }_{r}^{\ast }=100$, ${\beta }_{r}^{\ast }=5$, the blue one is ${\alpha }_{0}^{\ast }=50$, ${\alpha }_{r}^{\ast }=15$, ${\beta }_{r}^{\ast }=10$ and the red one is ${\alpha }_{0}^{\ast }=200$, ${\alpha }_{r}^{\ast }=10$, ${\beta }_{r}^{\ast }=20$. The (**b**) panel shows the overall cell population size, reflecting the average proliferation capacity while panel (**c**) shows the values embedding adverse effects of senescence, resulting in functionality drop.

Parameter *β*_0_ = *β*(*t*_0_) > 0 is the initial (or baseline) value, *β*_*r*_ > 0 the strength of the decline of cell functionality, and *t*_*d*_ > 0 is the time of death. Low *β*_*r*_ values will lead to a linear decline towards *t*_*d*_ after the age of peak performance. Conversely, high values show that the functional capabilities are preserved with aging until a sharp exponential decrease toward *t*_*d*_ occurs. Solving Equation (4) for *P*(*t*) yields:

7 $$P(t)={\beta }_{0}{N}_{0}\cdot {e}^{\frac{{\alpha }_{0}}{{\alpha }_{r}}(1-{e}^{-{\alpha }_{r}t})}\cdot (1-{e}^{{\beta }_{r}(t-{t}_{d})})$$

where *N*_0_ is the initial (or baseline) number of cells at *t*_0_. The right side of the equation is similar to the second component of the right side of Eq. (1) but includes *t*_*d*_ as the explicit time of death. Equation (7) can also be written as:

8 $$P(t)={\beta }_{0}{N}_{\infty }\cdot {e}^{-\frac{{\alpha }_{0}}{{\alpha }_{r}}{e}^{-{\alpha }_{r}t}}\cdot (1-{e}^{{\beta }_{r}(t-{t}_{d})})$$

with the cell population at the asymptote, ${N}_{\infty }={N}_{0}{e}^{\frac{{\alpha }_{0}}{{\alpha }_{r}}}$. This is a convenient formalization as it reduces the complexity of the multidimensional space and allows for a faster convergence in the search for optimal solutions using the Levenberg-Marquardt algorithm^34^. The reduced form of Eq. (8) is:

9 $$x(u)={e}^{-\frac{{\alpha }_{0}^{\ast }}{{\alpha }_{r}^{\ast }}{e}^{-{\alpha }_{r}^{\ast }u}}\cdot (1-{e}^{{\beta }_{r}^{\ast }(u-1)})$$

with $u\equiv \frac{t}{{t}_{d}},x(u)$ defining the dimensionless quantity $x(u)\equiv \frac{P(t)}{{\beta }_{0}{N}_{\infty }}$ and ${\alpha }_{0}^{\ast }\equiv {\alpha }_{0}{t}_{d}$, ${\alpha }_{r}^{\ast }\equiv {\alpha }_{r}{t}_{d}$, ${\beta }_{r}^{\ast }\equiv {\beta }_{r}{t}_{d}$. The behavior of this equation is pictured in Fig. 1 for different parameter values.

### Data collection and non-linear regressions

A set of 17 time series were collected from available on-line sources, previous publications and experimental studies:

- Physical performances in 8 track and field sport events. The speeds in m.s^−1^ are collected for the 100 m, 400 m, 800 m, 3000 m, 5000 m, 10000 m, marathon and the force momentum (in kg.m) for shot-put events in men. A total of 57587 performances were collected for the 100 m in the 1983–2013 period, 21955 for the 400 m (1970–2013 period), 17741 for the 800 m (1970–2013 period), and 21253 for the 5000 m (1970–2013 period). The shot put data (*n* = 11391) was converted to mass-distance in mass-meters (kg.m). Sources for cadet (15–16 years old), junior (17–18 years old) and elite series were found at http://www.iaaf.org and from previous personal data sets^10^. An additional source for younger ages is http://age-records.125mb.com/, and an additional source for elite athletes is on Tilastopaja (http://www.tilastopaja.org/). Sources for Master’s series (>35 years old) were collected from: http://www.world-masters-athletics.org (WMA). The best performance per age from the 3000 m to the 10000 m was found on the Association of Road Racing Statisticians: http://www.arrs.net/.
- Physical performances in weightlifting were collected from the International Weightlifting Federation (IWF, http://www.iwf.net/) and Master’s Weightlifting website (http://www.iwfmasters.net). A total of 2722 performances were collected.
- Chess performances in grand-masters: the individual careers of the first 96 chess players that show the greatest average ratings until 2011 were gathered, totaling *n* = 34481 ratings (source: http://www.chessmetrics.com/cm/). Chessmetrics ‘*weighted and padded simultaneous performance*’ ratings are used.
- Face recognition performance was collected for 44680 individuals who completed the Online Cambridge Memory Test. The proportion of correct answer per age was gathered from Fig. 3 in Germine *et al*.^15^.
- The racing speeds of thoroughbreds were collected from international 1200 m on-turf competition over a 10-year period (2005–2014) (source: http://jra.jp/). A total of 1810 speeds were gathered.
- Physical performance in greyhounds. A total of 26571 speeds (in m.s^−1^, for both males and females) were collected from http://www.greyhound-data.com. The entire careers of the 100 best individuals of the 480 meters (typical race distance) were gathered for each year over a ten-year period (2003–2012).
- The maximal distance per day in the wheel activity is recorded for mice (*Mus domesticus*) based on their voluntary behavior to practice wheel activity^35^. A total of 159 mice were genetically selected for high locomotor activity and 14241 daily wheel running performances (7078 for males and 7163 for females) were gathered^36^. The distance run was converted to km.week^-1^.
- The maximal grip strength of mouse lemurs (M*icrocebus murinus*) was measured using a small iron bar mounted on a piezo-electric force platform (Kistler squirrel force plate, ±0.1 N; Winterthur, Switzerland), connected to a charge amplifier (Kistler charge amplifier type 9865). The pull strength was recorded during 60 s at 1 kHz. Animals performed the pull task several times during this interval. The task consisted of letting the animal grab the iron bar and pull them horizontally until they let it go. The maximum force was then extracted using the Bioware software (Kistler). The repeatability of this task was previously verified^37^. A sample of 359 individuals was measured: 170 females and 189 males, with 39 individuals tested twice. A total of 398 grip-strengths were measured.

The convex envelop was determined by gathering the top performance at each age in each time series. The chosen time discretization is years of life for all traits measured in humans, months for traits measured in thoroughbreds and greyhounds, and weeks for traits measured in mice and mouse lemurs. All ages were converted to years of life and speeds are converted in m.s^−1^, except for the mice in km.week^−1^. Both Eqs (1) and (8) were then fit to each time series, including the two forces events, using a non-linear regression. The Levenberg-Marquardt nonlinear least squares algorithm was used to provide the estimated parameters in both equations. It is a typical algorithm to solve non-linear least squares problems^38^. Typical goodness-of-fit indicators were estimated^39^ (Tables 1 and S3 and Supplementary Materials).

**Table 1:** Adjusted *R*² for both Eq. (1) (Moore) and Eq. (8) (IMAP1) along with the estimated age of peak performance (year).

Time series	*R*² (Moore)	*R*² (IMAP1)	Peak (year) Moore	Peak (year) IMAP1
100 m (m.s^−1^)	0.9915	0.9924	25.88	24.99
400 m (m.s^−1^)	0.9819	0.9854	25.46	22.70
800 m (m.s^−1^)	0.9833	0.9848	25.74	25.40
3000 m (m.s^−1^)	0.9715	0.9742	23.53	28.24
5000 m (m.s^−1^)	0.9752	0.09815	25.65	28.95
10000 m (m.s^−1^)	0.9726	0.9803	25.74	30.15
Marathon (m.s^−1^)	0.9736	0.9764	27.82	26.94
Shotput (kg.m)	0.9174	0.9715	24.42	23.88
Weightlifting Clean & Jerk (kg)	0.9551	0.9757	24.72	31.39
Chess (score)	0.9781	0.9786	31.39	30.76
Facial recog. (proportion correct)	0.9501	0.9558	29.61	30.06
Greyhound (m.s^−1^)	0.9208	0.9207	23.31	22.18
Mouse males (km.week^−1^)	0.8801	0.8801	0.14	0.11
Mouse females (km.week^−1^)	0.9172	0.9163	0.15	0.15
Mouse lemur males (N)	0.0423	0.0330	1.53	3.59
Mouse lemur females (N)	0.0899	0.0843	4.87 × 10^−2^	2.06
Thoroughbred (m.s^−1^)	0.3981	0.3998	5.25	4.58

### Credibility Interval (CI)

A Monte-Carlo simulation was used to define a credibility interval in Eqs (1) and (8). The estimated covariance matrix for the fitted parameters was estimated for each non-linear regression. A total of 100,000 new parameters were then drawn from a multi-dimensional normal distribution that uses this matrix. The two convex envelopes that contain all these iterations are presented for each equation (see Supplementary Materials).

### Time constants *τ*

In order to further investigate the biological scope of the model, we compared the time constants *τ* provided by the model (1/*α*_*r*_) with the ones existing in the growth series. The primary assumption is that the total number of cells *N* roughly corresponds to the body mass (kg) of the studied species. We collected data from the official growth curves of the national French men population for humans and estimated the growth curves from the mouse lemur cohort and a thoroughbred database (see Supplementary Materials, Table S4 and Fig. S2).

## Results

**Figure 2:**
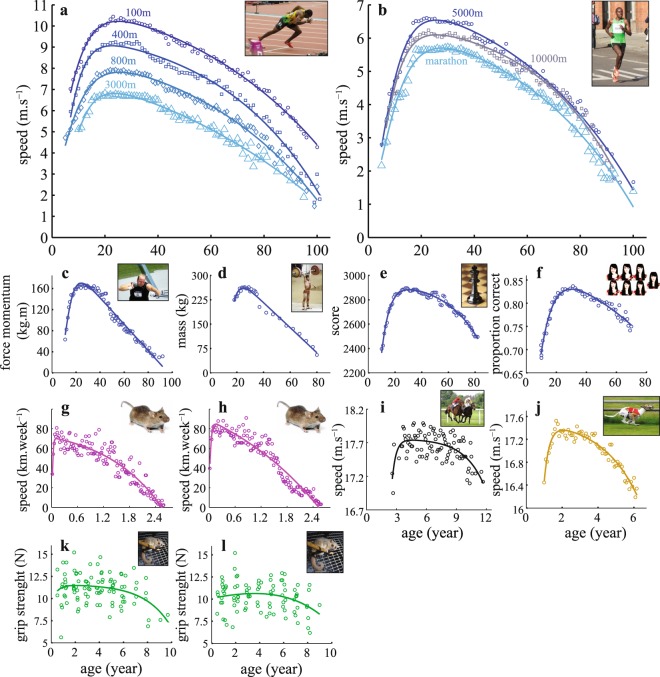
IMAP1 fitting in 17 events. Blue curves correspond to human systems (**a**–**f**), light purple one to mouse (**g**,**h**), black one to the thoroughbred (**i**), brown one to greyhound (**j**), green one to the mouse lemur (**k**,**l**). (**a**) Men sprint events, from the 100 m straight (blue dots) to the 3000 m (blue triangles) in T&F. (**b**) Men long-distance running events, from the 5000 m (blue dots) to the marathon (blue triangles). (**c**) Is the shot put (men), (**d**) is the clean & Jerk weight lifting (105+ kg, men), (**e**) is the chess event, (**f**) is the result from the Cambridge face memory test event. (**g**,**h**) Are the weekly speed for male and female mice respectively. (**i**) Is the speed of the thoroughbreds (1200 m turf competitions). (**j**) Is the greyhound speed in 480 m races (males & females). (**k**,**l**) Are the grip strength for the male and female mouse lemurs respectively. Inset images in (**a**,**c**) are licensed under the Creative Commons Attribution 2.0 Generic license (https://creativecommons.org/licenses/by-sa/2.0/), inset image in (**e**) is licensed under the Creative Commons Attribution-Share Alike 2.5 Generic license (https://creativecommons.org/licenses/by-sa/2.5/), inset images in (**b**,**d**,**f**,**i**–**l**) are licensed under the Creative Commons Attribution-Share Alike 3.0 Unported license (https://creativecommons.org/licenses/by-sa/3.0/) and inset images in (**g**,**h**) are in the public domain. Inset images are available at the following URLs: (**a**) commons.wikimedia.org/wiki/File:London_2012_Yohan_Blake_200m_Q.jpg; (**b**) commons.wikimedia.org/wiki/File:Patrick_Makau_Musyoki_running_world_record_at_Berlin_Marathon_2011.jpg; (**c**) commons.wikimedia.org/wiki/File:Adam_Nelson_2010_Outdoors.jpg; (**d**) commons.wikimedia.org/wiki/File:RIAN_archive_103479_Soviet_weight-lifter_Viktor_Mazin_during_the_XXII_Olympic_Games.jpg; (**e**) commons.wikimedia.org/wiki/File:Chess_piece_-_Black_king.JPG; (**f**) commons.wikimedia.org/wiki/File:Universal_emotions7.JPG; (**g**) commons.wikimedia.org/wiki/File:Mouse_white_background.jpg; (**h**) commons.wikimedia.org/wiki/File:Mouse_white_background.jpg; (**i**) commons.wikimedia.org/wiki/File:Horse-racing-4.jpg; (**j**) commons.wikimedia.org/wiki/File:Greyhound_Racing_4_amk.jpg; (**k**) commons.wikimedia.org/wiki/File:Gray_Mouse_Lemur_1.JPG; (**l**) commons.wikimedia.org/wiki/File:Gray_Mouse_Lemur_1.JPG.

**Figure 3:**
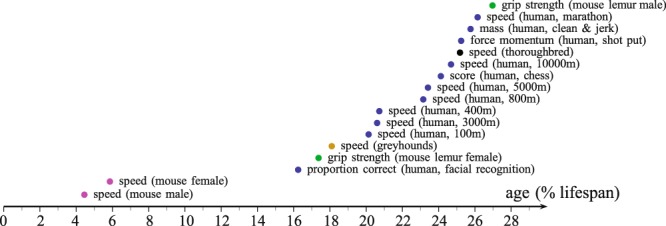
Normalized age of peak performance. It is computed as a percentage of estimated lifespan value: $peak/{t}_{d}$.

The age-related patterns are similar for the studied time series, revealing an inverted-U shape with continuous transitions between development and senescence (Fig. 2). The IMAP1 model provides good estimates of the performance on all time series (Fig. 2, Table 1, Supplementary Table S3). The normalization nevertheless reveals a few differences (Table 2). Estimated coefficients differ widely in the strength series (shotput and weightlifting), and in the mouse systems compared to other time series. They reveal a linear decline (small ${\beta }_{r}^{\ast }$ and high ${\alpha }_{0}^{\ast }$), possibly related to missing data at advanced ages and/or small cohorts introducing large variability. The lack of data during the steep decline is also quite important in the facial recognition time series, thus limiting the estimation of *t*_*d*_. In all studied systems, the age of peak performance occurs before the first half of the overall lifespan. The average normalized value of the age of peak performance is: $peak/{\tilde{t}}_{d}$ where ${\tilde{t}}_{d}$ is the estimated age of death. The peak performance is 20.49 ± 6.58% (Fig. 3).

**Table 2:** Estimated parameters of Eq. (8), including *t*_*d*_, *α*_*r*_ and the estimated time constant *τ* for humans, mice, mouse lemurs, greyhounds and thoroughbreds.

Time series	${\alpha }_{0}^{\ast }$	${\alpha }_{r}^{\ast }$	${\beta }_{r}^{\ast }$	*t*_*d*_ (year)	1/*α*_*r*_ (year)	*τ* (year)
100 m (m.s^−1^)	34.26	21.40	2.19	124.13	5.80	2.07, 9.21, **2**.**17** (*)
400 m (m.s^−1^)	67.64	25.51	2.79	109.54	4.29	/
800 m (m.s^−1^)	25.29	16.92	2.18	110.01	6.50	/
3000 m (m.s^−1^)	62.50	22.00	1.45	114.20	5.19	/
5000 m (m.s^−1^)	45.24	19.25	2.20	109.51	5.69	/
10000 m (m.s^−1^)	54.46	20.96	3.24	104.27	4.98	/
Marathon (m.s-1)	34.32	16.00	2.27	106.39	6.65	/
Shotput (kg.m)	223.84	20.28	3.01 × 10^−4^	96.73	4.77	/
Weightlifting Clean & Jerk (kg)	1011.39	25.70	3.74 × 10^−4^	95.96	3.73	/
Chess (score)	32.31	23.61	5.10	127.54	5.40	/
Facial recog. (proportion correct)	22.37	25.09	2.36	185.00	7.37	/
Greyhound (m.s^−1^)	77.83	39.96	5.64	11.37	0.28	N/A
Mouse males (km.week^−1^)	402.31	159.20	1.91	2.55	1.60 × 10^−2^	[0.19, 0.25] (*)
Mouse females (km.week^−1^)	108.53	83.74	0.97	2.53	3.02 × 10^−2^	[0.37, 0.67] (*)
Mouse lemur males (N)	0.66	0.39	2.03	13.31	33.88	2.24
Mouse lemur females (N)	5.01	25.80	5.57	11.84	0.46	2.24
Thoroughbred (m.s^−1^)	1125.04	46.33	9.16	18.21	0.39	1.06

*Note*. N/A = Not Available; (*) see Supplementary Materials and Table S4 for details.

### Model comparison

Based on the previously defined goodness-of-fit indicators and the credibility interval, the equation that best describes the age-performance relationship is the proposed one (Table 1 & S3 and Fig. S1): mean *R*^2^ = 0.85 ± 0.29 (IMAP1) vs. 0.83 ± 0.28 (Moore) and mean RMSE = 2.54 ± 4.51 (IMAP1) vs. 3.79 ± 6.54 (Moore). The corrected Akaike information criterion shows that the model is the best candidate among the two, except in 6 time series: greyhounds speed, both mouse lemur series and thoroughbred speed (Table S3). The differences are small, putting forward a similar fit for the two equations in this series. The dynamic time warping distance shows that the two equations are close in almost all cases, except in the two force events (shotput and weightlifting), chess rating and mice performance. Both equations do not suffer of over-parameterization and suggest that second order polynomial and quadratic curve fitting will be less efficient in both describing the age-performance relationship and providing an estimate of the age of peak performance. The CIs show that the IMAP’s ones are narrower and converge as the age increase due to the explicit age of death parameter *t*_*d*_. In Moore’s equation, the CIs diverge as the age increases (Fig. S1), due to the lack of such a parameter.

## Discussion

Aging research has widely focused on underlying mechanisms leading to observed declines in physical and physiological capabilities. Many studies describe the decline in locomotor speed, perceptual or cognitive functions, components of memory, reaction time, nerve conduction speed, muscular strength and flexibility, and other traits, and show that experimental investigations are valuable because they provide information about the dynamics of changes during the lifespan. In particular aging effects are studied in human sporting events^9,11,12,22,25,40^ where it is non-linear with time and has a declining curved shape. The extension of such research to other species may also help in identifying the common physiological and biological features that are responsible for this decline^4,26^. Other studies report a similar pattern while describing the decline in physical or physiological capacities in mammals^26^, insects^26^ and even in some plants^41^. Thoroughbreds and greyhounds have historical records in racing competitions. Data is very limited however: Racing horses are usually retired when they are 5 or 6 years old (4 or 5 years old for greyhounds) because of health, attitude and performance issues.

The IMAP1 Eq. (8) is based on a coupled 2-phase approach of aging effects describing an increase in organism’s capabilities at younger ages and a global impairment at older ones. Two parameters are used to give possible explanations in performance’s decline: the replicative senescence *α*(*t*) and the alteration in cellular effectiveness *β*(*t*). The latter is related to a range of causes which are still under investigation. Possible candidates include persistent metabolic waste products, interaction between damaged cell components (e.g., misfolded proteins), reactive oxygen species, telomere attrition, etc^42^. Faced with the many theories proposed to explain aging -ranging from the oxidative stress theory of aging to the antagonistic pleiotropy hypothesis^43^- we simply assume that the cellular functionality experience cumulative stresses during aging.

Moore’s equation has been frequently used to describe changes in physical or cognitive performance with aging^10,25,27^. A difference between Moore’s equation and the model presented herein is the additional parameter *t*_*d*_ that corresponds to an explicit time of death, where the decreasing process reaches 0. The parameter *t*_*d*_ is introduced in the IMAP1 and allows for both a controlled convergence and reduced variability near the time of death (see the Monte-Carlo credibility interval analysis in the Supplementary Materials and Supplementary Fig. S1). Moreover, the results show that estimated *t*_*d*_ are good approximations of empirical lifespan in each species (Table 2). Although a minor correction in the model, it remains an important formalization step as it is coupled to the developmental phase. The model can be adjusted to other physiological traits or species using any typical statistical software that implements non-linear regression algorithms. The results provided in Table S1 can also be useful to set the initial conditions of the algorithms (i.e. the initial values of the 4 parameters).

The IMAP1 allows for an accurate description of the studied physiological and physical events (Tables 1 and 2, Fig. 2 and Supplementary Table S3). While the physiological and biological processes implicated in those time series may widely differ, they all exhibit a similar pattern. It also shows that the convex envelop of top performances per age is congruent in different species, complementary to a previous work by Marck *et al*.^26^. The estimated time constants are in the same order of magnitude as the ones estimated for growth curves, with a few exceptions, of which mice are the most notable example (Table 2 and Supplementary Materials). It suggests that the model can to some extent describe the link between cellular proliferation and performance dynamics, despite its overall simplicity. It also strengthens the performance-body mass relationship in the developmental stage, where an increase in mass leads to an increase in speed to a certain extent. The observed discrepancy in mice would suggest that the impact of body mass on voluntary wheel-running activity is limited; however, several important factors need to be considered. First, body mass was a strong predictor of voluntary wheel running in the original selection experiments^44^. Second, body mass showed a negative correlated response to selection on voluntary wheel running, indicating a negative genetic covariance between voluntary activity and body^35^. Third, the mouse data presented here are from a population that experienced 16 generations of directional selection for high voluntary wheel running activity, meaning these mice are genetically different from the original source population of “normal” lab mice^35^. Nonetheless voluntary wheel-running activity may be weakly correlated with maximum speed, as it may rely on other mechanisms that explain the difference, such as motivation and coordination^26,44^.

The age of peak performance occurs at early ages (Fig. 3, Table 1) and all patterns exhibit a positive, right skewed profile (Fig. 2). It suggests that the senescent phase is longer than the developmental one. In the model, the detrimental processes start at the very initial stage of life but are compensated by the rapid growth of the organism in size (Eq. (5)). The initial locomotor capabilities of most terrestrial species at the earlier ages tend to be limited but not null: horse or zebra foals begin to walk anywhere from 45 minutes to 120 minutes after birth and wildebeest can stand up and move a few minutes after birth^45^. Slight differences appear when looking at the parameters (Table 2): both male and female mice have a higher ${\alpha }_{r}^{\ast }$ than the other time series. It is known that female mice mature earlier than males but display a later peak of activity than males^46^. Accordingly, the estimated ages of peak activity are 8.1 weeks (females) and 5.9 weeks (males).

Complex changes in structure and function occur at all levels of biological organization, from cells to whole body systems, eventually leading to the performance drop in a cumulative fashion^47–49^. This fits with previous observations in which the decline in physical performance with aging was described as highly non-linear^12,26^. It appears that maintaining a constant capacity at advanced ages is impossible and would require more and more energy. The mice and strength series have a smaller ${\beta }_{r}^{\ast }$ than most other species, revealing a linear decline. This may be related to a lack of or difficulties in implementing efficient exogenous artificial strategies (*i*.*e*., medical interventions) that delay aging effects. Such strategies exist in human-related systems, including greyhounds and thoroughbreds, where modern medical knowledge plus technological innovations slow the detrimental effects of aging on performance. From a broader view, it would suggest that the inverted-U shape pattern and ${\beta }_{r}^{\ast }$ values are related to the primary energy input^50^: the more energy is used to slow the decline, the higher ${\beta }_{r}^{\ast }$, although such an idea has yet to be investigated. But this could explain why the resulting patterns have a more parabolic declining shape in systems subject to medical intervention. The two strength events also present a linear decline, suggesting that it is difficult to offset strength loss with age, contrary to traits such as distance running. The amount of data at the extremes of aging is crucial, especially in organisms that exhibit a sharp pattern of growth toward maturity or a sharp decline toward death. In the first case, the developmental stage is very brief so that it is difficult to gather sufficient amounts of experimental data. The initial shape of the pattern is vague and this can lead to crude estimates of the age of peak performance and *α*_*r*_. The mouse and mouse lemur are particularly affected by this issue. Likewise, the lack of data in the declining part of the lifespan can lead to flawed estimates of β_r_. This is the case in the human facial recognition time series where it results in a widely overestimated *t*_*d*_ of 185 years old, which also leads to an inconsistent normalized estimate in the age of peak performance (*i*.*e*. 16.25% of estimated lifespan for a peak age of 30.06 years old). Correcting the value of *t*_*d*_ to *t*_*d*_ = 122 years old -the official maximum lifespan value ever recorded- in Eq. (8), does not dramatically change the age of peak performance (*i*.*e*. 30.63 years old) but produces a more normalized age of peak performance: 25.11%.

## Conclusion

A common inverted-U pattern is found for a range of physiological traits in different species. As compared with previous approaches, the proposed model accurately describes the age-related changes across the lifespan and is based on an enhanced biological foundation. The approach provides consistent credibility intervals, in favor of a finite lifespan with a non-linear decline of top performances at advanced ages. Additional investigations, targeting an accurate measurement of both parameters *α*_*r*_, *β*_*r*_, should allow for integrating endogenous biological stresses that increase with aging, such as extra-metabolic energy (*i*.*e*. independent of the calories derived from food resources only) and environmental factors that may alter declining strength. However, beside its approximation of the complex nature of the phenomenon, the IMAP1 provides a reasonable estimate of the age-performance relationship. In addition, in the future the model can be made more sophisticated, such as including cell specialization and explicit cell renewal which could possibly increase the model’s biological accuracy and lead to a better estimate of late-age performance decline.

## Electronic supplementary material

Supplementary Information
