# Supplementary material for: An integrative modeling approach to the age-performance relationship in mammals at the cellular scale
Source: Sci Rep. 2019 Jan 23;9:418. doi: 10.1038/s41598-018-36707-3 (PMC6344496; doi:10.1038/s41598-018-36707-3)
Supplement: Supplementary file 1 — Supplementary Information [file 41598_2018_36707_MOESM1_ESM.pdf]

An integrative modeling approach to the age-performance relationship in mammals at the cellular scale

Geoffroy Berthelot[1,2,*], Avner Bar-Hen[3], Adrien Marck[1,4], Vincent Foulonneau[1], Stéphane Douady[4], Philippe Noirez[1], Pauline B. Zablocki Thomas[5,6], Juliana da Silva Antero-Jacquemin[1], Patrick A. Carter[7], Jean-Marc Di Meglio[4], and Jean-François Toussaint[1,8]

[1]Institut de Recherche bio-Médicale et d'Épidémiologie du Sport (IRMES), EA 7329, Institut National du Sport, de l'Expertise et de la Performance (INSEP) and Université Paris Descartes, Sorbonne Paris Cité, Paris, France
[2]REsearch LAboratory for Interdisciplinary Studies (RELAIS), Paris, France
[3]CNAM, 75003 Paris, France
[4]Laboratoire Matière et Systèmes Complexes, UMR 7057, Université Paris Diderot and CNRS, Sorbonne Paris Cité, Paris, France
[5]Département de Biologie, ENS de Lyon, Lyon, France
[6]Département d'écologie et de Gestion de la Biodiversité, UMR 7179 CNRS/MNHN, Paris, France
[7]School of Biological Sciences, Washington State University, Pullman, USA
[8]CIMS, Hôtel-Dieu, APHP, Paris, France

Monday 12th November, 2018

* Corresponding author. geoffroy.berthelot@insep.fr, +33.41.74.41.86

In these supplementary notes, we provide additional information regarding the fitting operations and goodness-of-fit indicators. We detail some technical aspects of the Monte-Carlo procedure used for the credibility interval estimation. We also detail the time constants estimation from the growth curves.

Fitting operations

All fitting operations are performed with MATLAB R2011b (v. 7.13.0.564).

Coefficients estimates

Both the IMAP1 (eq. 8) and Moore's equations (eq. 1) are adjusted to the time series using the Levenberg-Marquardt algorithm. The estimated coefficients are provided in tables S1 and S2.

Time series	$\beta_0 N_\infty$ (same as $P(t)$)	α_0 (year^{-1})	α_r (year^{-1})	β_r (year^{-1})	t_d (year)
100m (m.s^{-1})	12.64	0.28	0.17	1.77×10^{-2}	124.13
400m (m.s^{-1})	10.31	0.62	0.23	2.55×10^{-2}	109.54
800m (m.s^{-1})	9.90	0.23	0.15	1.98×10^{-2}	110.01
3000m (m.s^{-1})	10.29	0.55	0.19	1.27×10^{-2}	114.20
5000m (m.s^{-1})	8.23	0.41	0.18	2.01×10^{-2}	109.51
10000m (m.s^{-1})	6.78	0.52	0.20	3.11×10^{-2}	104.27
Marathon (m.s^{-1})	7.25	0.32	0.15	2.13×10^{-2}	106.39
Shotput (N.m)	8.07×10^{5}	2.31	0.21	3.12×10^{-6}	96.73
Weightlifting Clean&Jerk (kg)	9.95×10^{5}	10.54	0.27	3.90×10^{-6}	95.96
Chess (score)	2.96×10^{3}	0.25	0.19	4.00×10^{-2}	127.54
Facial recog (proportion correct)	0.98	0.12	0.14	1.28×10^{-2}	185.00
Greyhound (m.s^{-1})	17.56	6.85	3.52	4.96×10^{-1}	11.37
Mouse male (km.week^{-1})	83.56	157.92	62.49	7.50×10^{-1}	2.55
Mouse female (km.week^{-1})	142.63	42.88	33.09	3.48×10^{-1}	2.53
Mouse lemur males (N)	62.60	4.98×10^{-2}	2.95×10^{-2}	1.53×10^{-1}	13.31
Mouse lemur females (N)	11.61	0.42	2.18	4.70×10^{-1}	11.84
Thoroughbred (m.s^{-1})	17.76	61.78	2.54	5.03×10^{-1}	18.21

Table S1 – Estimates of IMAP1 parameters.

Goodness-of-fit indicators

The residual sum of squares RSS is computed as:

$$\mathrm{RSS} = \sum_{i=1}^{n} (y_i - \tilde{y}_i)^2$$

Where y_i is the observation (the performance value) and $\tilde{y}_i$ the value estimated by the model. The root mean squared error RMSE is:

$$\mathrm{RSME} = \sqrt{\frac{1}{n}\mathrm{RSS}}$$

The corrected Akaike information criterion (AIC) is defined as:

$$\mathrm{AIC} = n \log\left(\frac{\mathrm{RSS}}{n}\right) + 2k + \frac{2k(k+1)}{n-k-1}$$

Where k is the number of parameters of the model: $k = 4$ for Moore's equation and $k = 5$ for the IMAP1 model. The bayesian information criterion (BIC) is:

$$\mathrm{BIC} = n \log\left(\frac{\mathrm{RSS}}{n}\right) + k \log(n)$$

Time series	a (same as $P(t)$)	b (year^{-1})	c (same as $P(t)$)	d (year^{-1})
100m (m.s^{-1})	11.40	0.14	1.74	1.61×10^{-2}
400m (m.s^{-1})	9.92	0.15	0.83	2.32×10^{-2}
800m (m.s^{-1})	8.83	0.13	1.17	1.95×10^{-2}
3000m (m.s^{-1})	8.50	0.11	6.25	7.23×10^{-2}
5000m (m.s^{-1})	7.85	0.11	1.81	1.50×10^{-2}
10000m (m.s^{-1})	6.82	0.12	0.48	2.57×10^{-2}
Marathon (m.s^{-1})	7.07	9.28×10^{-2}	1.59	1.57×10^{-2}
Shotput (N.m)	280.98	6.95×10^{-2}	8.87×10^{5}	3.38×10^{-6}
Weightlifting Clean&Jerk (kg)	406.34	8.83×10^{-2}	8.85×10^{5}	4.92×10^{-6}
Chess (score)	2.95×10^{3}	0.16	23.18	3.73×10^{-2}
Facial recog (proportion correct)	0.85	0.17	1.06×10^{-2}	3.37×10^{-2}
Greyhound (m.s^{-1})	17.53	2.90	8.32×10^{-2}	4.56×10^{-1}
Mouse male (km.week^{-1})	71.33	40.54	12.57	7.45×10^{-1}
Mouse female (km.week^{-1})	88.45	32.55	53.06	3.88×10^{-1}
Mouse lemur males (N)	10.59	5.31	1.08×10^{-2}	6.03×10^{-1}
Mouse lemur females (N)	11.44	253.06	2.34×10^{-2}	5.35×10^{-1}
Thoroughbred (m.s^{-1})	17.80	1.35	5.39×10^{-3}	4.15×10^{-1}

Table S2 – Estimates of Moore parameters.

BIC tends to select simpler models than the AIC.

The dynamic time warping is an algorithm that compares two signals that may be of different sizes. It uses a matrix valuated by the distance between each element of the two signals. We use it to compare the difference between the two fits in each time series. The distance between each element $\tilde{y}_i$ of the resulting fit of equation (1) and (8) is computed over the ageing interval $I = [t_1, t_1 + \delta, t_1 + 2\delta, \ldots, t_1 + (n-1)\,\delta, t_n]$, where t_1, t_n are the ages of the first and last observations respectively. The value $\delta = (t_n - t_1) \,/\, (M - 1)$ means that each equations are evaluated over M values from t_1 to t_n. We arbitrary set $M = 100$ as a convenient value for comparing the two fits. We then gather the 'path' through the matrix which minimizes the total distance between the two fits of equation (1) and (8). A window of size $w = 1$ is used to compute the distance of each element in the matrix.

The results are provided in tables S3.

Credibility interval

Definition

Let A be the estimated covariance matrix for the fitted parameters $\overrightarrow{X} = \begin{bmatrix} \widetilde{\beta_0}\widetilde{N}_\infty \\ \ldots \\ \widetilde{t_d} \end{bmatrix}$ of the equation $P(t)$ in a given time series. We use $i = 1, \ldots, n$, $n = 100000$ iterations of the equation $P_i(t)$ whose parameters values $\overrightarrow{X}_i$ are drawn from a multivariate normal distribution with distribution parameters $\begin{bmatrix} \left(\widetilde{\beta_0}\widetilde{N}_\infty, A\right) \\ \ldots \\ \left(\widetilde{t_d}, A\right) \end{bmatrix}$. The credibility interval C_t at a given age t corresponds to the boundaries defined by the maximal and minimal simulated values: $C_t = [\min P_i(t), \max P_i(t)]\,, \forall i$. The set of boundaries C, $\forall t$ is the maximal and minimal convex envelop of all the iterations.

Time series	RSS Moore	RSS IMAP1	RMSE Moore	RMSE IMAP1	AIC Moore	AIC IMAP1	BIC Moore	BIC IMAP1	DTW
100m ($m.s^{-1}$)	1.8213	1.6151	0.1431	0.1347	-337.65	-346.10	-328.18	-334.38	0.26
400m ($m.s^{-1}$)	5.5632	4.4135	0.2529	0.2252	-230.74	-248.63	-221.36	-237.04	0.92
800m ($m.s^{-1}$)	3.9932	3.5881	0.2083	0.1975	-280.16	-287.77	-270.54	-275.85	0.23
3000m ($m.s^{-1}$)	2.7821	2.4868	0.1966	0.1858	-225.65	-231.42	-217.14	-220.95	0.61
5000m ($m.s^{-1}$)	3.3434	2.4638	0.2019	0.1733	-253.86	-276.62	-244.75	-265.38	0.89
10000m ($m.s^{-1}$)	2.7863	1.9763	0.1821	0.1534	-277.61	-304.20	-268.39	-292.81	0.77
Marathon ($m.s^{-1}$)	3.3417	2.9482	0.1927	0.1810	-287.93	-296.96	-278.40	-285.18	0.61
Shotput (N.m)	1.18×10^4	4.01×10^3	12.7101	7.4151	379.78	303.41	388.35	313.97	92.82
Weightlifting Clean&Jerk (kg)	4.22×10^3	2.19×10^3	12.5040	9.0079	146.22	131.55	149.59	135.18	55.83
Chess (score)	2.57×10^4	2.48×10^4	18.7646	18.4167	436.66	436.23	445.23	446.79	22.11
Facial recog (proportion correct)	0.0039	0.0034	0.0084	0.0078	-536.77	-532.21	-519.46	-523.28	0.02
Greyhound ($m.s^{-1}$)	0.5370	0.5284	0.0923	0.0916	-154.81	-165.31	-152.20	-162.75	0.13
Mouse male ($km.week^{-1}$)	7.53×10^3	7.47×10^3	7.4972	7.4677	548.20	549.31	559.48	563.33	5.81
Mouse female ($km.week^{-1}$)	7.52×10^3	7.54×10^3	7.5746	7.5874	538.81	541.42	550.00	555.32	4.29
Mouse lemur males (N)	232.3129	231.9064	1.5891	1.5877	93.68	95.76	103.31	107.67	1.10
Mouse lemur females (N)	274.7580	273.5032	1.6744	1.6706	109.46	111.23	119.37	123.51	1.08
Thoroughbred ($m.s^{-1}$)	2.5407	2.5042	0.1671	0.1659	-317.17	-316.25	-307.59	-304.40	0.25

Table S3 – Goodness-of-fit indicators.

Limitations

The procedure is limited to the events which hold a reliable and large set of individuals at extreme age bands, such as the human-related ones. Otherwise:

- The increasing and / or declining shape is not well described and the estimated credibility interval (CI) can be large. Indeed, the unbounded search for an optimal solution in the multidimensional space using the Levenberg-Marquardt algorithm can lead to large values in A.
- The performance decreases in a linear or concave-up (with negative slopes) fashion, suggesting that it may smoothly approach 0 at ultimate ages and $\lim_{t \to \infty} P(t) = 0$. This may be due to both missing data -i.e. lack of individuals performing- and strong heterogeneity at these ages, resulting in a possible biased or insufficient description of the declining shape. It is formalized as an exponential decline in the two equations with parameters d (eq. (1)) and β_r (eq. (6) and (8)) strongly acting upon the estimated shape of $P(t)$, thus $\partial P(t)/\partial d$ (eq. (1)) and $\partial P(t)/\partial \beta_r$ (eq. (8)) may be large. Indeed, the 2-norm condition number of the estimated Jacobian matrix exceed the computer precision in the questioned time series, leading to infinite values in A.

For these two reasons, we focused in computing C_t in the following events: 100m, 400m, 800m, 5000m, 10000m, marathon and chess (Supplementary Figure S1).

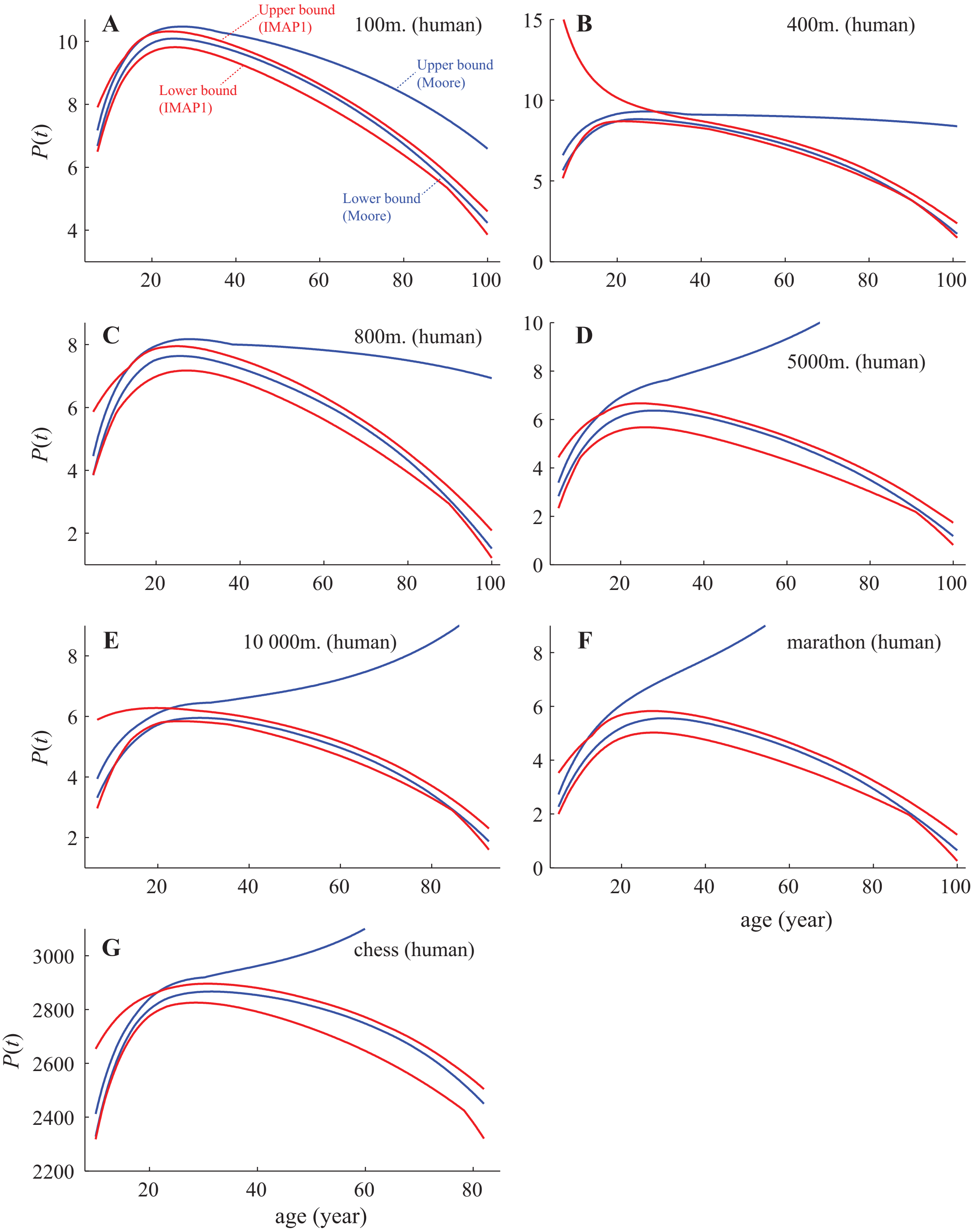

Figure S1 – CIs in 7 events (**A**-**G**). Each couple of lines represents the upper and lower boundaries containing all simulated iterations (see CI definition). The couples of red lines correspond to the IMAP1, while the blue ones correspond to the Moore equation.

Time constants estimates

All time constants τ are derived from growth curves in each of the studied species. The equation:

$$m = a\left[1 - e^{-b(t-c)}\right] \tag{10}$$

is adjusted to each series presenting a single birth-to-adult growing stage. The parameters a, b, and c are positive constants that are estimated through a non-linear regression (Levenberg-Marquardt algorithm) and m is the mass (kg). The time constants are provided by $\tau = 1/b$ for each series.

Humans

The human series presents a 3-stages 'ABC' development and we thus adjusted one particular equation to each of these stages:

$$\begin{aligned} m_A &= a\left[1 - e^{-b(t-c)}\right], && 0 \leq t < 5 && : \text{infancy / early childhood} \\ m_B &= ae^{bt}, && 5 \leq t < 14 && : \text{childhood / puberty} \\ m_C &= a\left[1 - e^{-b(t-c)}\right], && 14 \leq t < 22 && : \text{adolescence / adulthood} \end{aligned} \tag{11}$$

We focus on stage C that is closely related to performance development.

Mice

Bronikowski et al. measured the body mass in each of the four groups for males and females [1]. A total of 30171 body masses are gathered (160 males for a total of 15095 masses and 159 females for a total of 15076 masses). We focus on the first 70-80 weeks of data in order to estimate the time constants using equation 11. Results are showed in table S4 and we use the two intervals $[0.19, 0.25]$ (males), $[0.37, 0.67]$ (females).

Group	males	females
active control	0.2168	0.5520
active selection	0.2447	0.6705
sedentary control	0.1900	0.3710
sedentary selection	0.2282	0.4031

Table S4 – Estimates of τ for the 4 groups of mice.

Mouse lemurs

The body weight (kg) of 72 individuals, that participated in the grip strength experiment are gathered. The dataset consists in 31 males (1937 measures) and 41 females (3059 measures). We use equation 11 and resulting fits are presented in figure S2. The time constant are remarkably consistent for both genders: 2.2429 (males) vs. 2.2414 (females).

Thoroughbreds

Kocher and Staniar measured the growth pattern in 2184 horses (35044 weight measurements) and pointed out that the long term pattern of growth was similar to that in previous studies of thoroughbreds [2, 3]. We estimate τ using equation 11 on the data. Males and females are not set apart in this analysis because both genders appear in competition and establish similar marks.

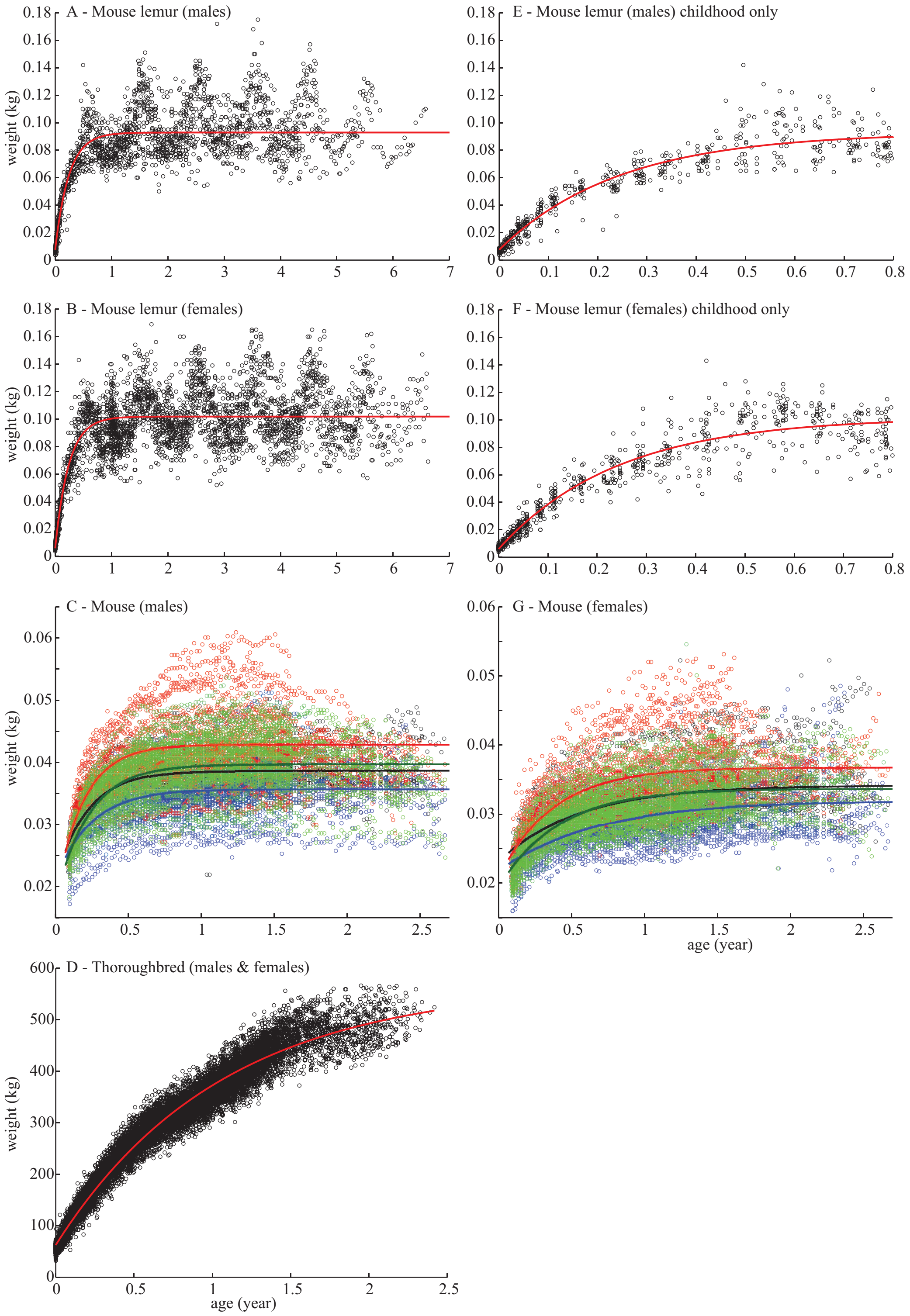

Figure S2 – Eq. 11 is adjusted to the body weight data in Mouse lemur (A & E, B & F), in the four groups of mice (black: active control, blue: active selection, red: sedentary control, green: sedentary selection) and in foals (C).

References

[1] AM Bronikowski, TJ Morgan, T Garland, and PA Carter. The evolution of aging and age-related physical decline in mice selectyively bred for high voluntary exercise. *Evolution*, 60(7):1494–1508, 2006.

[2] Andrea Kocher and W Burton Staniar. The pattern of thoroughbred growth is affected by a foal's birthdate. *Livestock Science*, 154(1):204–214, 2013.

[3] WB Staniar, DS Kronfeld, KH Treiber, RK Splan, and PA Harris. Growth rate consists of baseline and systematic deviation components in thoroughbreds. *Journal of animal science*, 82(4):1007–1015, 2004.
